# Role of the Transcriptional Repressor Zinc Finger with KRAB and SCAN Domains 3 (ZKSCAN3) in Retinal Pigment Epithelial Cells

**DOI:** 10.3390/cells10102504

**Published:** 2021-09-22

**Authors:** Hsuan-Yeh Pan, Mallika Valapala

**Affiliations:** School of Optometry, Indiana University, Bloomington, IN 47405, USA; hsupan@iu.edu

**Keywords:** autophagy, lysosomal function, zinc finger with KRAB and SCAN domains 3 (ZKSCAN3), retinal pigment epithelium (RPE)

## Abstract

Lysosomes are important for proper functioning of the retinal pigment epithelial (RPE) cells. RPE cells have a daily burden of phagocytosis of photoreceptor outer segments (POS) and also degrade cellular waste by autophagy. Here, we identified the role of Zinc-finger protein with KRAB and SCAN domains 3 (ZKSCAN3) in co-ordinate regulation of lysosomal function and autophagy in the RPE. Our studies show that in the RPE, ZKSCAN3 is predominantly nuclear in healthy cells and its nuclear expression is reduced upon nutrient deprivation. siRNA-mediated knockdown of ZKSCAN3 results in de-repression of some of the ZKSCAN3 target genes. Knockdown of ZKSCAN3 also resulted in an induction in autophagy flux, increase in the number of functional lysosomes and accompanied activation of lysosomal cathepsin B activity in ARPE-19 cells. We also demonstrated that inhibition of P38 mitogen-activated protein kinase (MAPK) retains ZKSCAN3 in the nucleus in nutrient-deprived cells. In summary, our studies elucidated the role of ZKSCAN3 as a transcriptional repressor of autophagy and lysosomal function in the RPE.

## 1. Introduction

The retinal pigment epithelium (RPE) is a monolayer of cells that performs several crucial functions vital for maintaining retinal homeostasis. RPE cells are responsible for the proper functioning of photoreceptor cells by phagocytosing 10% of their volume daily [1]. RPE cells are the most active phagocytic cells in the body as they phagocytose hundreds of thousands of outer segments in an individual’s lifetime [1]. Since, the RPE cells are post-mitotic, they are highly vulnerable to age-related accumulations of metabolic byproducts. Autophagy, a cellular degradation pathway, is crucially important to remove damaged structures and metabolic byproducts and reduce cellular accumulation in the RPE [2]. Dysfunctional autophagy process is known to result in impairment of RPE homeostasis, leading to age-related macular degeneration (AMD) [2]. During autophagy, cellular material and damaged organelles are sequestered in a double membrane organelle called autophagosome [3]. A crucially important stage in the autophagy process is the fusion of autophagosomes with lysosomes to form autophagolysosomes, where the autophagy substrates are degraded by the lysosomal hydrolases [4]. Since lysosomes are important in the terminal stages of the autophagy process, impaired lysosomal function can have significant implications on the autophagy pathway [3]. The effective clearance of the autophagy substrates is highly dependent on the functional ability of the lysosomal degradation pathway. In addition to being terminal organelles in the autophagy pathway, lysosomes are also known to directly regulate the autophagy pathway by influencing the activity of mTORC1 [5]. Several studies have implicated lysosomal dysfunction in the pathogenesis of AMD [6,7]. Age-related decline in lysosomal function is known to result in accumulation of phagocytosed outer segments and autophagic cellular substrates within the RPE, leading to impaired RPE function [6,7]. Acidification of the phagosome is also important for its proper degradation [8]. A2E, the major component of lipofuscin, is known to decrease the activity of the lysosomal proton pump and elevate lysosome pH [9]. Furthermore, oxidized lipoproteins contribute to a decline in lysosomal function by inhibiting the activity of lysosomal proteolytic enzymes [10]. 

Since, lysosomes and autophagy play an undeniably interdependent role in the RPE, strategies to induce both lysosomal function and autophagy can have tremendous implications in enhancing cellular clearance in the RPE. Hence, it is important to identify cellular mediators that coordinately regulate autophagy and lysosomal function in the RPE. Recent studies have identified transcription factor EB (TFEB) as a cellular regulator of both lysosomal and autophagy pathway [11]. During stress, activation and nuclear shuttling of TFEB results in transcriptional induction of genes involved in several stages of the autophagy and lysosomal biogenesis pathway [12]. In addition to transcriptional activators of autophagy and lysosomal function, autophagy-mediated cellular clearance functions are also under the control of transcriptional repressors. ZKSCAN3 (Zinc finger protein with KRAB and SCAN domains 3) is a member of a family of proteins containing zinc finger, Kruppel-associated box (KRAB), and SCAN domains [13]. ZKSCAN3 possesses the C2H2 zinc finger motif and a KRAB domain [13]. The KRAB domain is a highly potent transcriptional repressor module [14]. The KRAB domain in ZKSCAN3 is responsible for the transcription repression functions by binding to repressor proteins, whereas the C2H2 zinc finger motifs confer DNA binding ability [14]. The notion that proteins containing KRAB domains are transcriptional suppressors has been recently challenged by studies identifying some KRAB domain proteins such as ZKSCAN3, which can also stimulate colorectal tumor progression [15]. ZKSCAN3 also possess a highly conserved leucine rich 84 residue SCAN domain at the amino-terminus. The SCAN domain mediates protein–protein interactions either by self-association or by binding to other proteins [16]. 

ZKSCAN3 was recently implicated as a critical player in the transcriptional repression of autophagy and lysosomal function [13]. Gene expression profiling studies show that ZKSCAN3 acts as a transcriptional suppressor of about 60 genes involved in the lysosome biogenesis/function and autophagy pathway [13]. In addition, silencing ZKSCAN3 is known to increase the number of lysosomes in the cell [13]. Previous studies have shown that starvation-induced activation of protein kinase C-mediated signaling pathway phosphorylates ZKSCAN3, leading to its translocation out of the nucleus and subsequent de-repression of ZKSCAN3-regulated genes [17]. Unbiased screening studies have identified ZKSCAN3-binding motifs in genes regulating angiogenesis, cell migration, and growth, including vascular endothelial growth factor (VEGF) and integrin β4 [18]. These studies, taken together, suggest that ZKSCAN3 belongs to a small subset of transcription factors that co-regulate autophagy and angiogenic pathways. The biological significance of ZKSCAN3 in the RPE is largely unknown. In our studies, we aimed to determine the role of ZKSCAN3 in transcriptional regulation of lysosomal and autophagy pathways in response to cellular stress in the RPE.

In this report, we investigated the role of ZKSCAN3 in the RPE and the underlying regulatory mechanisms that contribute to the de-repression of ZKSCAN3 target genes in response to cellular stress. Our results indicated that nutrient deprivation diminishes nuclear levels of ZKSCAN3 resulting in de-repression and activation of ZKSCAN3 target genes, leading to induction of lysosomal function and autophagy pathway. Our results also show that in the absence of cellular stress, silencing of ZKSCAN3 induces lysosomal function and autophagy. Our findings provide insights into ZKSCAN3-regulated transcriptional program in the RPE which could be explored to induce cellular clearance in the RPE. 

## 2. Materials and Methods

### 2.1. Cell Culture and Transfection

Adult Retinal Pigment Epithelial cell line-19 (ARPE-19) cells were cultured in the presence of DMEM/F12 with L-Glutamine and 15 mM HEPES (Gibco; Thermo Fisher Scientific, Grand Island, NY, USA) along with 10% Fetal Bovine Serum (Hyclone; GE Healthcare Life Sciences, Logan, UT, USA) and 1% Antibiotic-Antimitotic (Gibco; Thermo Fisher Scientific, Grand Island, NY, USA). ARPE-19 cells are widely used as in vitro models to study the function of the RPE. The data obtained from ARPE-19 cells was further substantiated in primary RPE cells, isolated from C57BL6/J and ATP-binding cassette, sub-family A, member 4 (ABCA4) knockout mice as described previously [6]. Primary mouse RPE cells and RPE choroid extracts were isolated from C57BL/6J mice according to the protocol approved by the Institutional Animal Care and Use Committee, Indiana University/School of Optometry and conformed to the ARVO Statement for the Use of Animals in Ophthalmologic and Vision Research. For starvation, the cells were cultured in Earle’s Balanced Salt Solution (EBSS) (Gibco; Thermo Fisher Scientific, Grand Island, NY, USA) with calcium and magnesium for 24–48 h.

### 2.2. Antibodies

Details of the primary antibodies used are provided in Supplementary Table S1.

### 2.3. Cell Viability Assay

ARPE-19 cells were seeded in 96-well plates and treated with various concentrations of SMARTPOOL ZKSCAN3 siRNA (25, 50, 100, μM) (Dharmacon, Lafayette, CO, USA) for 24, 48, 72, and 96 h. We used a range of concentrations and time points to evaluate dose and time-dependent effects of the siRNA on the viability of the RPE. The medium with different concentrations was then removed and cells were incubated with 20 µL of 3-(4,5-dimethylthiazol-2-yl)-2,5-diphenyl tetrazolium bromide (MTT) (5 mg/mL) for 4 h at 37 °C. The optical density was measured at 490 nm using a Microplate Reader (BMG Labtechnologies, Cary, NC, USA) after dissolving the formazan with 0.5% DMSO.

### 2.4. Immunoblotting

ARPE-19 cells were treated with EBSS (Gibco; Thermo Fisher Scientific, Grand Island, NY, USA) and ZKSCAN3 siRNA (Dharmacon, Lafayette, CO, USA) at 100 nM for 48 h. Total cell lysates were obtained by adding Radioimmunoprecipitation assay buffer (RIPA) lysis buffer. Protein concentration was measured by bicinchoninic acid (BCA) protein assay (Thermo Fisher Scientific, Grand Island, NY, USA). The Sodium dodecyl sulfate polyacrylamide gel electrophoresis (National Diagnostics, Atlanta, GA, USA) was used to detect protein and transferred onto polyvinylidene difluoride (PVDF) membranes (Millipore Sigma, Burlington, MA, USA). The membranes were block with 5% milk made by Tris-buffered saline with 0.1% Tween^®^ 20 detergent (TBST) at room temperature for an hour and incubated with primary antibodies overnight at 4 °C followed by incubating in secondary. The image Lab software (Bio-Rad Laboratories, Hercules, CA, USA) was used to detect the bands.

### 2.5. Nuclear and Cytoplasmic Extraction

ARPE-19 cells were seeded in 10 cm petri dish and cultured in the presence of EBSS (Gibco; Thermo Fisher Scientific, Grand Island, NY, USA). Cells were transfected with ZKSCAN3 siRNA (Dharmacon, Lafayette, CO, USA) at 100 nM for 48 h. The cells were harvested using NE-PER Nuclear and Cytoplasmic Extraction Reagent kit (Thermo Fisher Scientific, Grand Island, NY, USA) following the manufacturer’s instructions. The α-tubulin antibody was developed by Walsh, C and was obtained from the Developmental Studies Hybridoma Bank, created by the NICHD of the NIH and maintained at The University of Iowa, Department of Biology, Iowa City, IA 52242.

### 2.6. Immunostaining and Microscopy

ARPE-19 and mouse primary RPE cells were seeded in 8 chamber slide and treated with Earle’s Balanced salt solution (EBSS)(Gibco; Thermo Fisher Scientific, Grand Island, NY, USA) and SMARTPOOL ZKSCAN3 siRNA (Dharmacon, Lafayette, CO, USA) at a concentration of 100 nM for 48 h. After 48 h, the cells were fixed by 4% paraformaldehyde, permeabilized with 0.5% triton-x diluted in PBS, and blocked with blocking buffer (5% BSA and 0.5% Tween-20 in 1× PBS and 10% goat serum (MP biomedicals, Irvine, CA, USA) The primary antibody was incubated overnight at 4 °C. Next day, cells were treated with secondary antibody, which was added for 1 h at room temperature. The slide was mounted by Prolong Gold antifade reagent with DAPI (Life technologies, Carlsbad, CA, USA). Cells were imaged under a Zeiss microscope equipped with a camera (Axiocam Mrm; Carl Zeiss, Oberkochen, Germany).

### 2.7. Quantitative Real Time-PCR

RNeasy Mini Kit (QIAGEN, Hilden, Germany) was used to isolate RNA from ARPE-19 cells 48 h after transfection with the ZKSCAN3 siRNA. Then, 400 ng of RNA was converted to cDNA by RNA-to-cDNA Kit (Applied Biosystems, Waltham, MA, USA) using SsoAdvanced™ SYBR^®^ Green Supermix (Bio-Rad Laboratories, Hercules, CA, USA). Primer sequences are provided in Supplementary Table S2.

### 2.8. Cathepsin B Activity Assay

ARPE-19 cells were seeded on 8-well chamber slides and treated with EBSS (Gibco; Thermo Fisher Scientific, Grand Island, NY, USA) and ZKSCAN3 siRNA (Dharmacon, Lafayette, CO, USA) at 100 nM for 48 h. Cathepsin B activity was determined using the Magic Red Cathepsin B Assay Kit (Immunochemistry). Briefly, Cells transfected with ZKSCAN3 siRNA for 48 h were incubated with 1× Magic Red dye for 1 h at 37 °C, rinsed with PBS and mounted. Cells were imaged by a Zeiss LSM800 microscope (20× objective). Images were quantified using the ImageJ software (NIH, Bethesda, MD, USA).

### 2.9. Lysosomal Function Assay

ARPE-19 cells were seeded in 8 chamber slide and treated with EBSS (Gibco; Thermo Fisher Scientific, Grand Island, NY, USA) and ZKSCAN3 siRNA (Dharmacon, Lafayette, CO, USA) at 100 nM for 48 h. LysoTracker Green DND-26 (Invitrogen, Waltham, MA) was used for lysosomal function activity examination. After 48 h, the cell medium was replaced with prewarmed (37 °C) probe-containing medium and incubated for 1 h in a 37 °C incubator. The image was examined by EVOS FL Auto (Life Technologies, Carlsbad, CA, USA). The slide was later mounted by Prolong Gold antifade reagent with DAPI (Life Technology, Carlsbad, CA, USA) examined by Zeiss microscope equipped with a camera (Axiocam Mrm; Carl Zeiss, Oberkochen, Germany) again.

### 2.10. Quantification and Statistical Analysis

Quantification of LC3 puncta was performed using ImageJ software (NIH, Bethesda, MD, USA). Briefly, the corrected total fluorescence intensity was calculated after background subtraction and the puncta were quantified using analyze particles plugin on ImageJ. Data is represented as mean LC3 puncta ± standard deviation of three independent experiments. Cathepsin B immunostaining was quantified by measuring corrected total cell fluorescence (CTCF), which was obtained by subtracting the product of area of selected cell and mean fluorescence of background readings from integrated density of each cell using ImageJ. CTCF is represented as mean fluorescence intensity ± standard deviation. Densitometry was measured for each band and represented relative to the corresponding loading control using ImageJ software. qRT-PCR data is quantified using the comparative or ΔΔCt method. qRT-PCR data is represented as fold change in the expression of a gene of interest relative to the reference gene. All data is presented as mean ± standard deviation. For statistical analysis, student’s *t*-test (two tailed) and one-way ANOVA with Tukey post hoc multiple comparison was used. Statistical analysis was performed using the Graphpad prism software. 

## 3. Results

### 3.1. Nutrient Deprivation Reduces Nuclear Levels of ZKSCAN3

**Figure 1:**
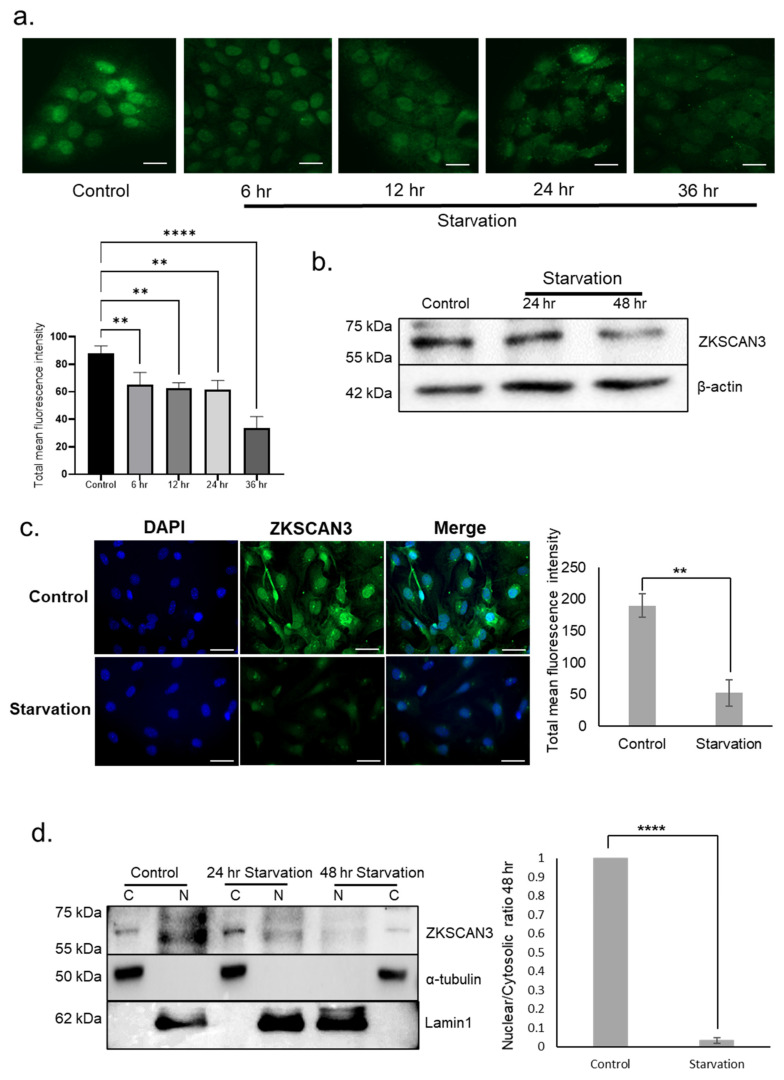
Nutrient deprivation results in reduced levels of ZKSCAN3 expression. (**a**) Immunostaining to determine ZKSCAN3 expression in ARPE-19; (**b**) immunoblot analysis of ZKSCAN3 expression in ARPE-19; (**c**) primary mouse RPE cells subjected to indicated periods of nutrient deprivation; (**d**) subcellular fractionation and ZKSCAN3 immunoblotting of cytosolic (C) and nuclear (N) fractions in cells subjected to nutrient deprivation. Lamin A and α-tubulin were used as controls for nuclear and cytosolic extracts, respectively. All data are presented as mean ± standard deviation. One-way ANOVA and student’s *t*-test (two tailed) were used for analysis *p*-value. The *p*-value is stated as ** *p* < 0.01, **** *p* < 0.0001. Scale = 20 μM.

We tested whether nutrient deprivation has an effect on the expression of ZKSCAN3 in the RPE. Subcellular localization of ZKSCAN3 was analyzed in ARPE-19 and primary mouse RPE cells subjected to nutrient withdrawal by culturing in EBSS. Immunofluorescence analysis showed a striking reduction in nuclear levels of ZKSCAN3 at different time points (6–36 h) upon nutrient withdrawal in ARPE-19 cells (Figure 1a). Quantification of total fluorescence intensity showed a 1.4- (*p* ≤ 0.01) and 2.6- (*p* ≤ 0.0001) fold decrease in levels of ZKSCAN3 expression in cells subjected to nutrient deprivation for 24 and 36 h, respectively, compared to control cells. Immunoblot analysis showed a reduction in the expression of ZKSCAN3 in cells starved for 24 and 48 h compared to control cells (Figure 1b). In primary mouse RPE cells a 3.6-fold reduction (*p* ≤ 0.01) in ZKSCAN3 expression was observed upon nutrient withdrawal for 48 h compared to control cells (Figure 1c). Immunoblotting studies also revealed a decrease in expression of ZKSCAN3 in mouse RPE choroid extracts isolated from nutrient-deprived mice compared to controls (Supplementary Figure S1). Nuclear cytoplasmic fractionation followed by immunoblotting of ARPE-19 cells subjected to nutrient deprivation for 48 h showed a 30-fold marked reduction (*p* ≤ 0.0001) in nuclear levels of ZKSCAN3 (Figure 1d). These results suggest that nutrient deprivation results in decreased nuclear expression of ZKSCAN3. 

### 3.2. Knockdown of ZKSCAN3 Increases the Expression of ZKSCAN3-Regulted Genes

**Figure 2:**
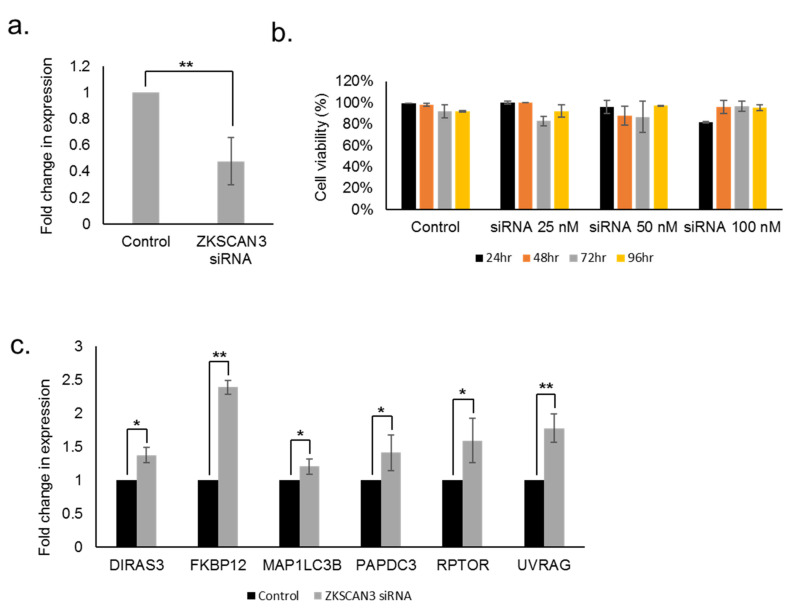
Expression of ZKSCAN3 and downstream targets upon knockdown of ZKSCAN3 in the RPE. (**a**) qRT-PCR analysis of ZKSCAN3 expression upon ZKSCAN 3 siRNA (**b**) Cell viability assay upon ZKSCAN3 siRNA treatment (**c**) qRT-PCR analysis of the following ZKSCAN3-regulated lysosomal autophagy genes. All data are presented as mean ± standard deviation. Student’s t-test (two tailed) was used for analysis *p*-value. The *p*-value is stated as * *p* < 0.05, ** *p* < 0.01.

Next, we investigated the cellular effects of ZKSCAN3 knockdown in ARPE-19 cells. Quantitative real time PCR (qRT-PCR) analysis revealed a 2.12-fold decrease (*p* < 0.01) in the expression of ZKSCAN3 upon siRNA knockdown (Figure 2a). To determine if ZKSCAN3 knockdown is a reasonable approach to stimulate autophagy, we first needed to show that reduced ZKSCAN3 expression did not affect cell viability. Our data shows that knockdown of ZKSCAN3 in a dose- and time-dependent manner had no effect on the viability of RPE cells (Figure 2b). Interestingly, knockdown of ZKSCAN3 in a variety of cancer cells resulted in induction of senescence [13]. These studies suggest that ZKSCAN3 knockdown modulated the functioning of key cellular processes such as the autophagy pathway in normal and cancer cells differently. Furthermore, expression of the following autophagy genes was enhanced in cells treated with ZKSCAN3 siRNA compared to control: DIRAS Family GTPase 3 (DIRAS3) (1.37-fold increase, *p* < 0.05), FK506 Binding Protein 12 (FKBP12) (2.38-fold increase, *p* < 0.01), Phosphatidic acid phosphatase type 2 domain-containing protein 3 (PPAPDC3) (1.41-fold increase, *p* = 0.05), Microtubule-associated protein 1A/1B-light chain 3 (MAP1LC3B) (1.20-fold increase, *p* < 0.05), UV Radiation Resistance Associated (UVRAG) (1.77-fold increase, *p* < 0.01). Surprisingly, we also observed an induction of Regulatory-associated protein of mTOR (RAPTOR) (1.59-fold increase, *p* < 0.05) in cells treated with ZKSCAN3 siRNA compared to control cells. These studies suggest that suppression of ZKSCAN3 enhances the expression of several key genes in the autophagy pathway (Figure 2c). 

### 3.3. Induction of Autophagy upon Silencing of ZKSCAN3

**Figure 3:**
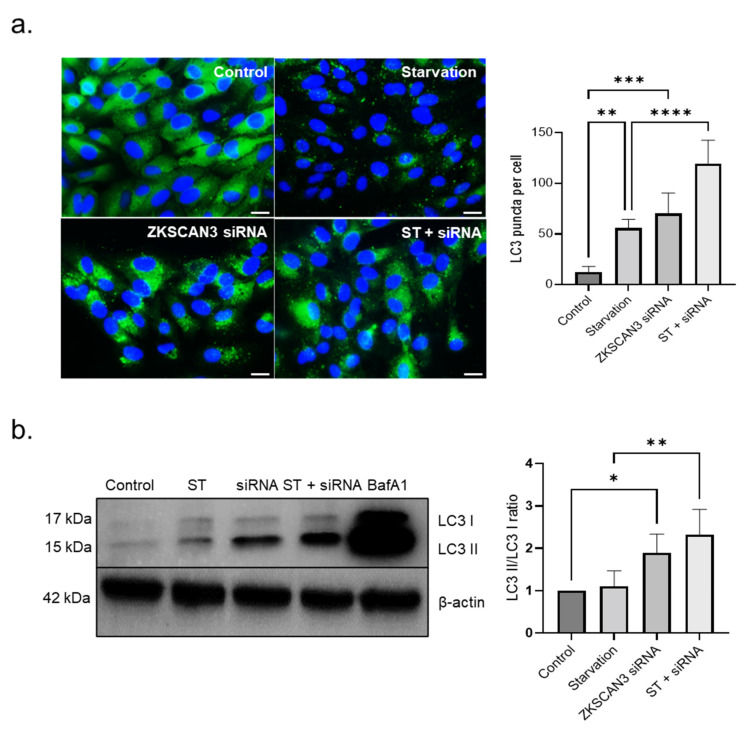
Knockdown of ZKSCAN3 induces autophagy in the RPE. (**a**) Immunostaining with LC3 antibody and quantification of LC3 puncta in cells treated with ZKSCAN3 siRNA compared with control. (mangified images are provided in Supplementary Figure S2) (**b**) LC3 immunoblotting of control, cells starved (ST), ZKSCAN3 siRNA treated (siRNA), siRNA-treated and starved cells (ST + siRNA) and bafilomycin A1 (BafA1)-treated cells. All data are presented as mean ± standard deviation. One-way ANOVA was used for analysis *p*-value. The *p*-value is stated as * *p* < 0.05, ** *p* < 0.01, *** *p* < 0.001, **** *p* < 0.0001. (ST indicates starvation). Scale = 20 μM.

Our results show that silencing ZKSCAN3 in ARPE-19 cells increased the number of LC3 puncta in both ZKSCAN3 siRNA transfected (5.75-fold increase, *p* < 0.001) and starved cells transfected with ZKSCAN3 siRNA (2.2- fold increase, *p* < 0.0001) compared to control and starved cells, respectively (Figure 3a). Next, we investigated the effect of ZKSCAN3 knockdown on autophagy flux by monitoring LC3I to LC3II conversion. Flux through the autophagy system is measured by comparing LC3II/LC3I ratio in the presence and absence of the lysosomal inhibitor bafilomycin A1, which prevents the degradation of LC3II. LC3II/LC3I ratio in cells transfected with ZKSCAN3 siRNA showed a 1.89-fold increase (*p* < 0.05) compared to control cells. Nutrient deprived cells transfected with ZKSCAN3 siRNA showed a 2.1-fold increase (*p* < 0.01) in LC3II/LC3I ratio compared to cells subjected to starvation alone. Moreover, the increase in LC3II/LC3I ratio upon ZKSCAN3 suppression is significantly lower compared to LC3II/LC3I ratio in the presence of Bafilomycin A1, which strongly suggests that ZKSCAN3 knockdown induces the autophagy pathway (Figure 3b). These results suggest that knockdown of ZKSCAN3 results in autophagy induction in the RPE.

### 3.4. Knockdown of ZKSCAN3 Induces Lysosomal Function in the RPE 

**Figure 4:**
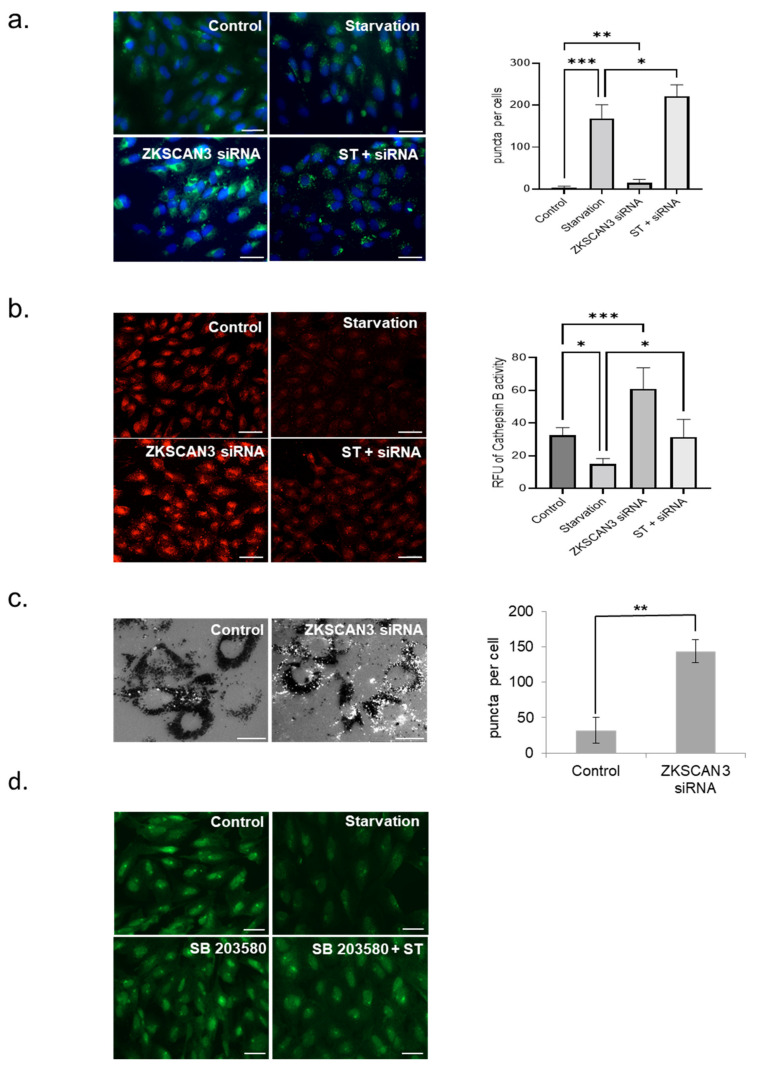
Knockdown of ZKSCAN3 induces lysosomal function in the RPE. (**a**) Staining with the LysoTracker dye in control and in cells ZKSCAN3 siRNA compared with control. Quantification of LysoTracker puncta using ImageJ software. Scale = 20 μM. (**b**) Measurement of Cathepsin B activity using the magic red cathepsin B substrate upon knockdown of ZKSCAN3. Quantification of cathepsin B staining using ImageJ software. Scale = 40 μM. (**c**) Staining with LysoTracker dye to evaluate lysosomal biogenesis in ABCA4 knockout RPE upon ZKSCAN3 siRNA knockdown for 48 h. Scale = 10 μM. (**d**) ZKSCAN3 immunostaining in the presence of P38 MAPK inhibitor, SB203580. Scale = 20 μM. All data are presented as mean ± standard deviation. One-way ANOVA and Student’s *t*-test (two tailed) were used for analysis *p*-value. The *p*-value is stated as * *p* < 0.05, ** *p* < 0.01, *** *p* < 0.001. (ST indicates starvation).

Next, we investigated the effect of ZKSCAN3 suppression on lysosomal function in the RPE. ARPE-19 cells transfected with ZKSCAN3 siRNA for 48 h were stained with a lysotracker dye to label lysosomes. LysoTracker probes are membrane permeant acidotropic dyes used for selective labeling of acidic lysosomes. Incubation of lysotracker probe for 1 h results in selective peri-nuclear labeling of lysosomes in cells transfected with ZKSCAN3 siRNA. Our results show that suppressing ZKSCAN3 expression increased the number of acidic lysosomes in cells cultured under normal conditions (3.7-fold increase, *p* < 0.01) compared to control cells. However, knockdown of ZKSCAN3 in nutrient-deprived cells showed a modest induction the number of functional lysosomes (1.3-fold increase, *p* < 0.05) compared to staved cells (Figure 4a). 

Since the lysotracker dyes label acidic vesicles, it can be inferred from our studies that knockdown with ZKSCAN3 siRNA increases the basal number of acidic lysosomes in the RPE cells. Lysosomal acidity is crucial to the degradative functions of lysosomes. Alkalization of lysosomes results in decreased lysosomal function, impairs processing of lysosomal substrates, and thereby cause cellular accumulation of undigested and partially digested material [19]. Lysosomal acidity is indispensable for the normal functioning of the lysosomal proteases and imbalances in lysosomal pH affects the activity of these degradative enzymes [20]. Since knockdown of ZKSCAN3 resulted in increased number of lysotracker-labeled acidic lysosomes, we measured if the increased acidic lysosomes result in a concomitant increase in the activity of the lysosomal enzyme, cathepsin B. The cathepsin B substrate, Magic Red easily penetrates the plasma membrane and organellar membranes in a non-fluorescent state and localizes to acidic organelles such as lysosomes. Upon entry into the lysosomes, Magic Red is cleaved by mature lysosomal Cathespin B to yield the cresyl violet fluorophore that fluoresces upon excitation. Since, lysosomal pH is crucial for the activity of cathepsin B, an increase in the number of acidic lysosomes or decrease in pH of the lysosomal compartment leads to enhanced activity of the Cathepsin B. Our results show that increase in the number of functional lysosomes in ZKSCAN3 siRNA-treated cells is also accompanied by a significant induction (1.88-fold increase, *p* < 0.001) in the enzyme activity of Cathepsin B, a lysosomal protease, compared to control (Figure 4b). In nutrient-deprived cells, treatment with ZKSCAN3 siRNA resulted in a 2.1-fold increase (*p* < 0.05) in cathepsin B activity compared to starved cells. In addition, we tested the effects of ZKCAN3 knockdown on ATP binding cassette subfamily A member 4 knockout (ABCA4 KO) RPE, which was previously shown to possess elevated pH in the lysosomes [21]. Our data shows that ZKSCAN3 siRNA treatment resulted in a significant increase (4.48-fold increase, *p* < 0.01) in the number of lysotracker-stained acidic lysosomes in the ABCA4 KO RPE compared with control (Figure 4c). In the fed state, ZKSCAN3 is present predominantly in the nucleus causing transcriptional repression of both autophagy and lysosomal genes. In the starved state, nuclear expression of ZKSCAN3 is diminished, leading to depression of ZKSCAN3-regulated genes. Previous studies showed that protein kinase C δ (PKCδ) acting through P38MAPK (Mitogen-activated protein kinase) phosphorylates ZKSCAN3, resulting in its nuclear export and alleviation of ZKSCAN3-mediated repression [17]. Our results show that in ARPE-19 cells treated with the P38 MAPK inhibitor, SB203580, ZKSCAN3 is retained in the nucleus even under conditions of nutrient deprivation (Figure 4d).

## 4. Discussion

In recent years, remarkable progress has been made in our understanding of the molecular events that coordinately regulate the transcription of genes involved in the lysosomal and autophagy pathway. It is also well known that lysosomes are crucial for the proper completion of the autophagy process, and unsurprisingly lysosomal function and autophagy are co-coordinately regulated by two major transcription factors; ZKSCAN3 (zinc-finger protein with KRAB and SCAN domains 3) and transcription factor EB (TFEB) [22,23]. TFEB and ZKSCAN3 are known to possess opposing actions, suggesting that they function as on and off switches, respectively, to regulate lysosome biogenesis/function and autophagy in response to cellular stress [23,24].

Age-related decline in lysosomal enzyme activity due to accumulation of A2E contributes to the progressive accumulation of cellular material in the RPE [7,9]. Autophagy serves as a protective mechanism against cellular stresses such as oxidative stress, lipofuscin accumulation, and mitochondrial dysfunction [4,25]. In this manuscript, we elucidated the protective role of lysosomal and autophagic enhancement mediated by ZKSCAN3 suppression in the RPE. ZKSCAN3 is a transcription suppression factor that exerts its biological effects by binding to repressor domains in target genes and inhibiting both autophagy and lysosomal processes [13]. Our studies show that in normal cells, ZKSCAN3 is predominantly nuclear and represses its target genes to maintain only basal levels of autophagy. When cells are stressed, nuclear levels of ZKSCAN3 are reduced, causing de-repression and induction of ZKSCAN3 target genes. SiRNA-mediated knockdown of ZKSCAN3 induces starvation-induced autophagy responses in ARPE-19 cells. We also observed an increase in acidic lysosomes and lysosomal Cathepsin B activity.

Furthermore, identifying the mechanisms that regulate the activity of ZKSCAN3 is critical to target ZKSCAN3 in order to induce autophagy and lysosomal function in the RPE. Previous studies showed that protein kinase C δ (PKCδ) acting through P38MAPK (Mitogen-activated protein kinase) phosphorylates ZKSCAN3, resulting in its nuclear export and alleviation of ZKSCAN3-mediated repression [17]. Our results show that ARPE-19 cells treated with the P38MAPK inhibitor, SB203580, retains ZKSCAN3 in the nucleus even under conditions of nutrient deprivation. In addition to the PKC pathway, the mTOR-signaling pathway is known to be associated with autophagy. mTOR is a key regulator of the autophagy pathway and is known to affect the activity of several autophagy pathway kinases by phosphorylation [26]. Inhibition of mTOR signaling by rapamycin has been used as a strategy to induce autophagy in order to promote cellular clearance in various aggregation-prone degenerative diseases [27]. In addition, PKCδ-dependent and mTOR-independent mechanisms of autophagy and lysosomal regulation are explored. Recent studies have also shown that PKC activators induce PKC-dependent export of ZKSCAN3 from the nucleus and also promote clearance of amyloid β (Aβ) plaques in mouse models of Alzheimer’s disease [17]. Our results support previous studies showing that activation of PKCδ through P38MAPK pathway can be used as a strategy to inhibit endogenous ZKSCAN3 in the RPE.

In summary, our studies have elucidated a role of the transcription suppressor, ZKSCAN3 in regulating autophagy and lysosomal function in the RPE. Our results have shown that knockdown of ZKSCAN3 induces autophagy and lysosomal function in the RPE. These studies have significant therapeutic potential as they lead to the identification of novel molecular targets that could be harnessed to induce cellular clearance processes in the RPE and protect RPE cell death. However, a recent study using a mouse model lacking ZKSCAN3 has shown that the expression of ZKSCAN3-regulated autophagy and lysosomal genes was not affected *in vivo* [14]. In mouse embryonic fibroblasts, loss of ZKSCAN3 did not result in an induction of autophagy upon nutrient withdrawal [14]. These conflicting results could be due to the differences in ZKSCAN3 amino acid composition between human and mouse proteins, which likely contributes to the functional differences in protein function. These responses could also be specific to our siRNA-mediated knockdown approach.

Since, TFEB and ZKSCAN3 have opposing functions [23], our future studies are directed towards elucidating reciprocal regulation of both the transcription factors under conditions of nutrient stress. We will also study whether changes in the expression of one transcription factor affects the expression of the other factor in the RPE. Furthermore, previous studies have shown that P38 MAPK regulates autophagy via the glycogen synthase kinase 3β (GSK3β) signaling pathway [28]. Since GSK3β also effects TFEB signaling [28], future studies are directed towards investigating the role of P38 MAPK pathway in co-regulation of TFEB and ZKSCAN3 in the RPE.

## Data Availability

The datasets generated in the manuscript will be made available from the corresponding author on reasonable request.

## Supplementary Materials

The following are available online at https://www.mdpi.com/article/10.3390/cells10102504/s1, Table S1: The details of primary antibodies, Table S2: The sequence of human primers, Figure S1: immunoblot analysis of ZKSCAN3 expression in mouse RPE tissue, Figure S2: Immunostaining with LC3 antibody in cells treated with ZKSCAN3 siRNA compared with control.
