# Supplementary material for: Role of the Transcriptional Repressor Zinc Finger with KRAB and SCAN Domains 3 (ZKSCAN3) in Retinal Pigment Epithelial Cells
Source: Cells. 2021 Sep 22;10(10):2504. doi: 10.3390/cells10102504 (PMC8534123; doi:10.3390/cells10102504)
Supplement: Supplementary file 1 [file cells-10-02504-s001.zip › cells-1349073-supplementary.pdf]

# Role of the transcriptional repressor Zinc Finger with KRAB and SCAN Domains 3 (ZKSCAN3) in retinal pigment epithelial cells

Hsuan-Yeh Pan and Mallika Valapala*
School of Optometry, Indiana University, Bloomington, IN

Mallika Valapala, Ph.D.
Indiana University School of Optometry
800 E. Atwater Avenue
Bloomington, IN 47401, USA
Email: mvalapal@iu.edu

Hsuan-Yeh Pan
Indiana University School of Optometry
800 E. Atwater Avenue
Bloomington, IN 47401, USA
Email: hsupan@iu.edu

## Supplementary tables

## Table S1: The details of primary antibodies

| Antibodies | Application | Vendor |
|---|---|---|
| anti-ZKSCAN3 antibody | WB: 1:1000 IF :100 | Abcam |
| anti-LC3 antibody | WB: 1:1000 IF 1:500 | MBL International |
| Purified anti-Lamin A Antibody | WB: 1:1000 | BioLegend |
| anti-β-Actin antibody | WB: 1:2000 | Sigma |
| anti-$\alpha$-tubulin antibody | WB: 1:5000 | Developmental Studies Hybridoma Bank |

## Supplementary tables

## Table S2: The sequence of human primers

| Human Primers | Sequence |
|---|---|
| DIRAS3-R | GAACAGCTCCTGCACATTCA |
| DIRAS3-F | CATAAGTTCCCCATCGTGCT |
| FKBP12-R | GTGGCACCATAGGCATAATCTGG |
| FKBP12-F | TGCTAGGCAAGCAGGAGGTGAT |
| PPAPDC3-R | GCCACCGTCATGATGTCCAG |
| PPAPDC3-F | TGGTCAAGCTCATCGGCATC |
| RAPTOR-R | TCAAGGCTCTGCTTGTACCG |
| RAPTOR-F | TCGATCCTCTGTCGATGGGT |
| MAP1LC3B-R | AGATTGGTGTGGAGACGCTG |
| MAP1LC3B-F | AGCAGCTTCCTGTTCTGGAT |
| UVRAG-R | GACGGTCTGGCATAATTCCAAA |
| UVRAG-F | GGCGTCTTCGACATCTTCGG |

# Supplementary figure

## Figure S1

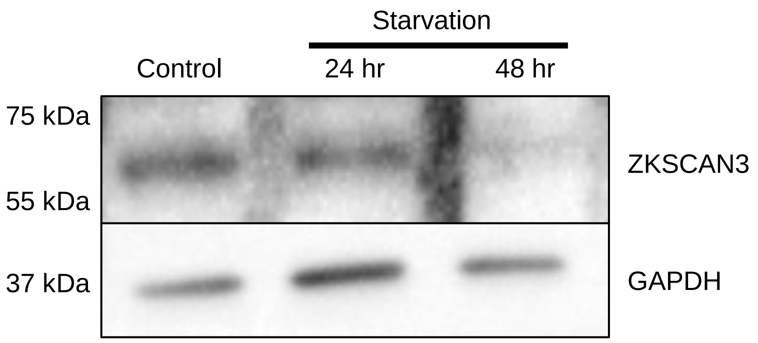


Figure S1: immunoblot analysis of ZKSCAN3 expression in mouse RPE tissue.

## Figure S2

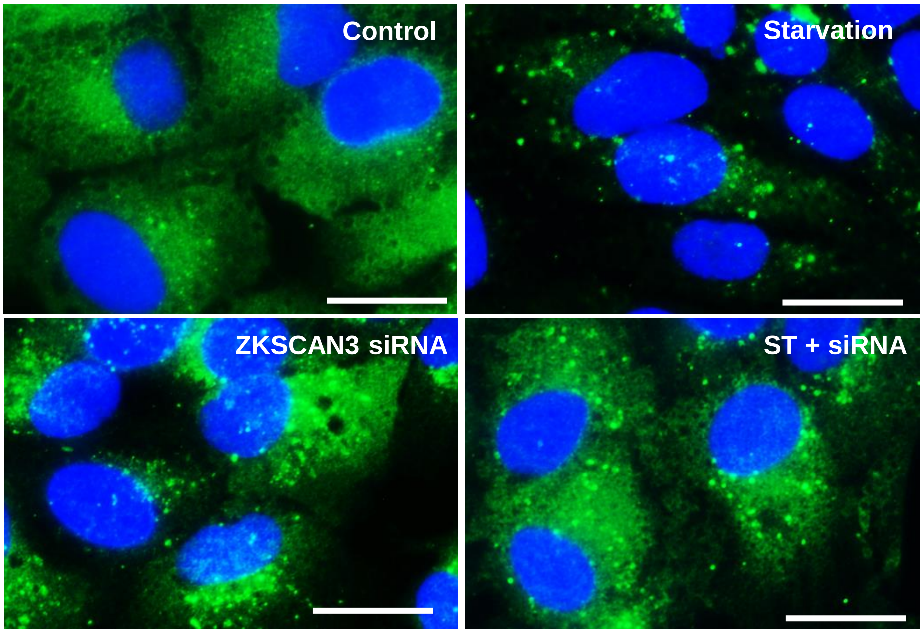


Figure S2: Immunostaining with LC3 antibody in cells treated with ZKSCAN3 siRNA compared with control. Scale =20 μM.
